# Clinical Criteria for Persistent Inflammation, Immunosuppression, and Catabolism Syndrome: An Exploratory Analysis of Optimal Cut-Off Values for Biomarkers

**DOI:** 10.3390/jcm11195790

**Published:** 2022-09-29

**Authors:** Kensuke Nakamura, Kentaro Ogura, Hiroyuki Ohbe, Tadahiro Goto

**Affiliations:** 1 Department of Emergency and Critical Care Medicine, Hitachi General Hospital, 2-1-1, Jonan-cho,, Hitachi 317-0077, Ibaraki, Japan; 2 Faculty of Medicine, The University of Tokyo, 7-3-1 Hongo, Bunkyo, Tokyo 113-8655, Japan; 3 TXP Medical Co., Ltd., 7-3-1 Hongo, Bunkyo, Tokyo 113-8485, Japan; 4 Department of Clinical Epidemiology and Health Economics, School of Public Health, The University of Tokyo, 7-3-1 Hongo, Bunkyo, Tokyo 113-0033, Japan

**Keywords:** PICS, critical care, inflammation, immunosuppression, catabolism

## Abstract

**Background:** While clinical criteria have been proposed for persistent inflammation, immunosuppression, and catabolism syndrome (PICS) using C-reactive protein (CRP), albumin, and lymphocyte count, there is no substantial basis for their optimal cut-off values. We herein aimed to develop and externally validate clinical criteria for PICS by investigating the optimal cut-off values for these biomarkers using machine-learning approaches and confirmed it with external validation. **Methods:** To develop criteria, we included ICU patients treated at a tertiary care hospital in Japan between 2018 and 2021 (derivation cohort). We introduced CRP, albumin and lymphocyte counts at around day 14 into six machine-learning models to predict PICS, defined as the compound outcome of the Barthel index (BI) < 70 at hospital discharge and in-hospital death. We incorporated the results of these models to assess the optimal cut-off values for biomarkers. We then developed and externally validated criteria for PICS using a nationwide claims database in Japan (validation cohort). **Results:** In the derivation cohort, 291 out of 441 patients had BI < 70 or in-hospital death. Based on machine-learning models, the optimal cut-off values for biomarkers to predict them were a CRP of 2.0 mg/dL, albumin of 3.0 g/dL, and a lymphocyte count of 800/μL, with an AUROC of 0.67. In the external validation cohort, 4492 out of 15,302 patients had BI < 70 or in-hospital death. The AUROC of the criteria was 0.71, with sensitivity of 0.71 and specificity of 0.68 to predict PICS. **Conclusions:** We herein provide a fundamental basis for PICS clinical criteria with CRP >2.0 mg/dL, albumin <3.0 g/dL, and a lymphocyte count <800/μL on day 14. The criteria developed will identify patients with PICS whose long-term mortality and activity of daily living may be poor.

## 1. Introduction

With advances in intensive care, the mortality of critically ill patients has markedly decreased and many patients admitted to intensive care units (ICU) survive [1]. However, chronic critical illnesses, in which patients remain in severe conditions and their hospital length of stay extends over a long time, represent another challenge [2,3,4]. Hyperinflammation, known as systemic inflammatory response syndrome SIRS, often occurs in severe patients, simultaneously accompanying a number of anti-inflammatory responses called as compensatory anti-inflammatory response syndrome CARS [5,6]. SIRS and CARS should normally converge with healing; however, they occasionally do not and prolong even after the acute phase of critical care. Many of these patients have a prolonged hyperinflammatory condition with body exhaustion and susceptibility to infection, which is called persistent inflammation, immunosuppression, and catabolism syndrome (PICS) [7,8]. The long-term prognosis of patients with PICS is poor in terms of mortality or quality of life [9]. Although the expansion of myeloid-derived suppressor cells (MDSCs) has been proposed as the main factor contributing to the development of PICS [10], this pathological condition is more complex between inflammation and immunosuppression [11].

The following clinical criteria have been proposed for PICS in the first literature of PICS [7,10]: prolonged hospitalization >14 days, C-reactive protein (CRP) >0.15 mg/dL as hyperinflammation, a total lymphocyte count <800/μL as immunosuppression, and albumin <3.0 g/dL, weight loss of >10% or body mass index <18 during hospitalization, a creatinine height index <80%, pre-albumin <10 mg/dL, or retinol-binding protein <10 μg/mL as catabolism. Although these biomarkers do not directly reflect catabolism or immunosuppression, their combination may facilitate the identification of patients with PICS. A previous study using machine-learning methods on the trajectory of CRP in ICU patients suggested that CRP converges at >3.0 mg/dL in patients with PICS [12]. Furthermore, the fundamental basis for the cut-off values for these biomarkers was not descried in the literatures [7,10] and has not yet been and established; therefore, it remains unclear whether these clinical criteria are appropriate for identifying patients with PICS.

Although PICS is primarily an immune dysfunction, functional decline has been emphasized with the need for long-term care and self-insufficiency [7]. Previous studies demonstrated that PICS or chronic critical illness is strongly associated with physical dysfunction after treatments [4,13]. The Barthel index (BI) is used to assess the activity of daily living (ADL), particularly the need of assistance [14]. BI of 60 to 80 appears to be the threshold for dependency [15,16,17]. Therefore, cut-off values for PICS biomarkers may be clinically evaluated by predicting the compound outcomes of lower BI and mortality in patients with long hospital stays.

We herein aimed to develop clinical criteria for PICS (defined as BI < 70 at discharge [18,19] or in-hospital death) by assessing optimal cut-off values for CRP, albumin, and lymphocyte count on day 14 after admission using machine-learning approaches. We then externally validated the criteria developed for PICS using a nationwide multicenter database. We also analyzed the data obtained using cut-off values of BI < 60 and <80 in the sensitivity analysis.

## 2. Materials and Methods

### 2.1. Study Design and Data Sources

This was a prognostic study using the data of the Hitachi General Hospital Emergency and Critical Care Center for the development of clinical criteria and Medical Data Vision (MDV) claims (Medical Data Vision Co., Ltd., Tokyo, Japan) to externally validate these criteria. Hitachi General Hospital is one of the largest tertiary medical centers in Japan, is located in a city with approximately three million residents, and has an annual ICU admission of approximately 2000 patients to an 18-bed closed mixed ICU. We used data obtained in the ICU/Emergency ward between March 2018 and January 2021. The MDV database contains electronic health insurance claims and Diagnosis Procedure Combination payment system data from more than 350 Japanese acute hospitals, covering more than 35 million patients as of 2020 (Medical Data Vision Co., Ltd.). Anonymized information is available on patient demographics, laboratory values, medical procedures, disease diagnoses, and inpatient and outpatient resource uses and costs. In the present study, we used MDV data between April 2014 and October 2020 for external validation. The present study was approved by the Institutional Review Board (IRB) of Hitachi General hospital, (2017-95) and the requirement for written informed consent was waived by the IRB.

### 2.2. Study Samples

Inclusion criteria were as follows:

1. Patients treated in the ICU, including postoperative patients
2. No long-term care insurance or home health care before admission.
3. Hospital stays >14 days.
4. BI recorded on the day of hospital discharge.

These inclusion criteria were used for criteria development using data from the Hitachi General Hospital Emergency and Critical Care Center. The criteria developed were externally validated using MDV data.

### 2.3. Measurements

Regarding patient characteristics, the following data were extracted and evaluated: patient demographics (e.g., age, sex, and comorbidities), the sequential organ failure assessment (SOFA) score, the acute physiology, chronic health evaluation (APACHE II) score on admission, and basic diseases recorded in electronic medical records. As adjunctive therapies, mechanical ventilation, renal replacement therapy, and the use of extracorporeal membrane oxygenation were extracted with their total duration (days) during admission. Due to data availability, the APACHE II score on admission was only calculated using data from Hitachi General Hospital.

### 2.4. Outcome Measurements

The primary outcome was PICS defined as the compound outcome of BI < 70 at hospital discharge [18,19] or in-hospital death. BI is scored between 0 and 100 based on the summed scores for 10 items: feeding, personal toileting, bathing, dressing and undressing, getting on and off a toilet, bladder control, bowel control, moving from a wheelchair to the bed and returning, walking on a level surface, and ascending and descending stairs [14].

## 3. Statistical Analysis

### 3.1. Derivation Cohort

To develop clinical criteria for PICS, we used CRP, albumin, and lymphocyte counts on day 14 after admission or on the nearest day to day 14 within days 11–17 after admission. In the case of missing laboratory data on days 11–17, we estimated data on day 14 using XGBoost, a non-parametric algorithm that accommodates for non-linearities and interactions without a particular parametric hypothesis for missing numerical parameters [20]. The variables used for the estimation are shown in Supplemental Table S1. Biomarker data were missing for CRP in 10.4% of cases, albumin in 14.1%, and lymphocyte count in 21.5%.

### 3.2. Evaluation of Optimal Cut-Off Values for CRP, Albumin, and Lymphocyte Count Using Machine-Learning Approaches

We employed linear regression, logistic regression, Gaussian naive Bayes, ridge regression, random forest, and XGBoost to assess optimal cut-off values for biomarkers. CRP, albumin and lymphocyte counts were introduced into the models and BI < 70 at hospital discharge or in-hospital death was assigned as the outcome to predict. Analyses were conducted using scikit-learn library ver. 0.24.1 and XGBoost library ver. 1.3.3 with Python ver. 3.8.7.

We searched for cut-off values using the following approach:

1. We initially selected the search range and intervals for each biomarker based on knowledge: CRP levels from a range of 0 to 4.0 mg/dL with an interval of 0.1 mg/dL, albumin levels from a range of 0 to 4.0 g/dL with an interval of 0.1 g/dL, and lymphocyte counts from 600 to 1400/μL with an interval of 20.
2. To search for the optimal cut-off values for biomarkers, we developed a machine-learning model by sequentially adding biomarkers to the model evaluated by area under the receiver operating characteristic (AUROC), which was calculated by five-fold cross-validation. We initially developed a CRP-only model and searched for the best AUROC by dichotomizing CRP from 0 to 4.0 mg/dL with an interval of 0.1 mg/dL. Once the best AUROC was identified, the corresponding CRP level was selected as the optimal CRP level. Using the optimal CRP level as a fixed value, we added albumin to the CRP-only model and searched for the best AUROC by dichotomizing albumin levels from 0 to 4.0 g/dL with an interval of 0.1 g/dL; the optimal albumin level was selected based on AUROC. Using the fixed optimal CRP and albumin levels, we then added lymphocyte count to the model and repeated the search process.
3. We repeated step #2 for each machine-learning model by changing the order in which we added biomarkers to the model (i.e., six patterns for each machine-learning model based on combinations of the orders of CRP, albumin, and lymphocyte count). Therefore, we developed 36 models to search for the optimal cut-off values for biomarkers.

### 3.3. Development of Criteria and Performance Evaluation

Using the 36 models, we examined the optimal cut-off values for CRP, albumin, and lymphocyte count based on their means, accounting for clinical usefulness and clinical consensus. We developed clinical criteria for PICS using optimal cut-off values as follows: albumin and lymphocyte count were given one point for being less than the cut-off value, while CRP was given one point for being greater [7,10]. Therefore, the criteria ranged between 0 and 3 points. Sensitivity and specificity were calculated to evaluate the predictive ability of each score of the developed criteria. We also generated Kaplan–Meier survival curves for 90-day in-hospital mortality and performed Log-rank tests between the scores of the developed criteria.

### 3.4. External Validation

To evaluate the generalizability of the clinical criteria developed, they were externally validated using MDV data. Only patients who had a complete data set for CRP, albumin, and lymphocyte count around day 14 after admission (i.e., on day 14 or on the nearest day between days 11–17) were included for external validation.

### 3.5. Sensitivity Analysis

To gain insights into the relationships between biomarker levels and PICS, we initially used the six best models for each machine-learning model to assess the optimal cut-off values for biomarkers. We then repeated the primary analysis for BI < 60 and <80 as an outcome of PICS.

## 4. Results

### 4.1. Study Flow and Patient Characteristics of the Derivation Cohort

**Figure 1:**
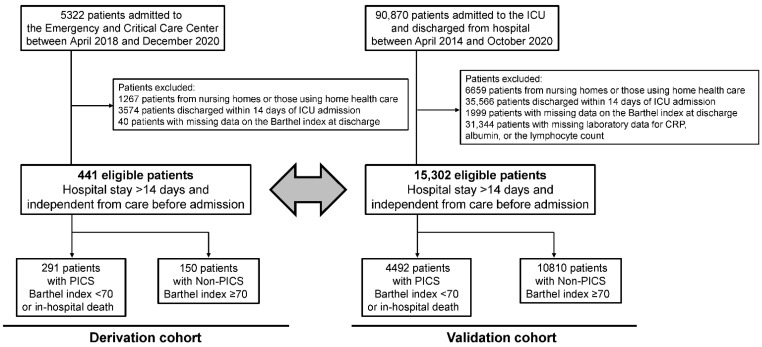
Patient flow. Abbreviations: ICU, intensive care unit; CRP, C-reactive protein; PICS, persistent inflammation, immunosuppression, and catabolism syndrome; Non-PICS, non-persistent inflammation, immunosuppression, and catabolism syndrome.

During the study period, 5322 patients were admitted to the Emergency and Critical Care Center. After exclusion, 441 eligible patients with a hospital stay >14 days and independent from care before admission were included in the present study as the derivation cohort (Figure 1). Among these patients, there were 291 patients with PICS (BI < 70 at hospital discharge or in-hospital death), and 150 patients with Non-PICS (BI ≥ 70 at hospital discharge). Patient characteristics and outcomes are shown in Table 1. The median age of patients was 71 years and 64.0% were male. Median SOFA and APACHE II scores were 6 and 17. Approximately 30% of patients had sepsis, followed by renal failure, endocrine and metabolic disorder, cardiac failure, and trauma. The overall mortality rate was 22.7%, and BI at hospital discharge was 55 (IQR, 10–100). Patients with PICS were more likely to be older, have a more severe condition and stroke, and have lower albumin on day one and higher CRP and lower albumin and lymphocyte counts on day 14 than those with Non-PICS.

### 4.2. Evaluation of Optimal Cut-Off Values and Development of Clinical Criteria

**Figure 2:**
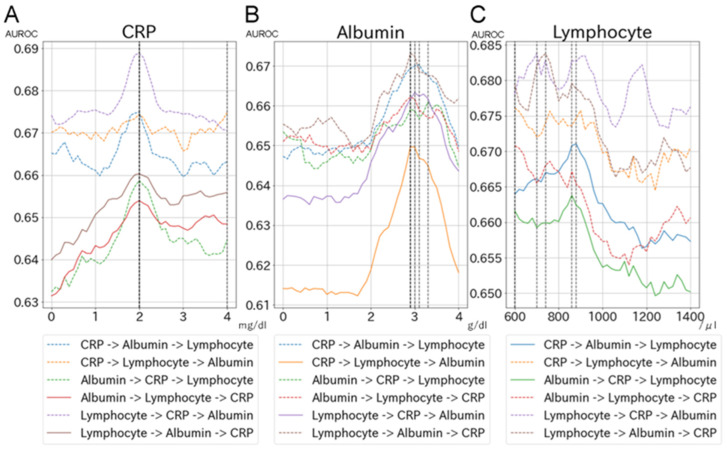
Changes in AUROC for each combination of the order of biomarkers in the XGBoost model. Figures show the relationship between each biomarker and the AUROC for the primary outcome using the XGBoost model: panel (**A**) indicates the change in the AUROC of CRP when the cut-off values of the other two biomarkers were fixed. Similarly, panels (**B**,**C**) indicate changes in the AUROCs of albumin and lymphocyte count when the cut-off values of the other two biomarkers were fixed. Each panel contains the results of six combinations of the order of biomarkers. Abbreviations: AUROC, area under the receiver operating characteristic; CRP, C reactive protein.

**Figure 3:**
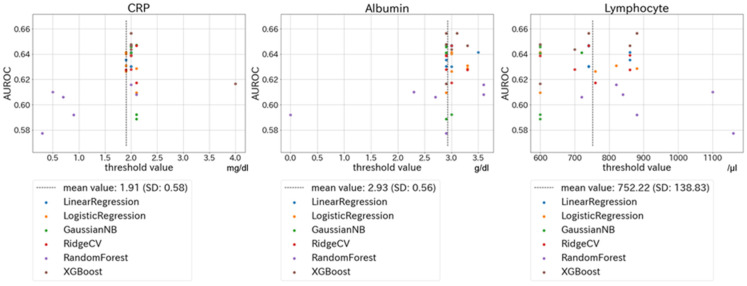
Scatter plots of cut-off values for biomarkers and corresponding AUROCs in 36 models. Figures show scatter plots of cut-off values for biomarkers in 36 models (i.e., six machine-learning models and six combinations of the order of biomarkers). The threshold values on the horizontal axis represent the best results for each model and order. Abbreviations: AUROC, area under the receiver operating characteristic; CRP, C reactive protein.

The relationship between each biomarker (CRP, albumin, and lymphocyte count) and AUROC for PICS (BI < 70 at discharge or in-hospital death) is shown in Figure 2 and Supplemental Figure S1. Figure 2 shows the relationship between each biomarker and AUROC for PICS using the XGBoost model: panel A indicates the change in the AUROC of CRP when the cut-off values of the other two biomarkers were fixed. Similarly, panels B and C show changes in the AUROCs of albumin and lymphocyte count when the cut-off values of the other two biomarkers were fixed. Figure 3 shows scatter plots of cut-off values for biomarkers in 36 models (i.e., six machine-learning models and six combinations of the order of biomarkers). Similar optimal cut-off values were suggested by all the models. The mean value for CRP levels in the 36 models was 1.91 mg/dL (SD, 0.58), while those for albumin and lymphocyte count were 2.93 g/dL (SD, 0.56) and 752/μL (SD, 139), respectively. Based on these results, we selected a CRP of 2.0 mg/dL, albumin of 3.0 g/dL, and a lymphocyte count of 800/μL as the optimal cut-off values for PICS.

The predictive ability of the criteria developed using the above cut-off values for PICS is shown in Table 2. The criteria ranged between 0 and 3 points, with an AUROC of 0.67. For example, the sensitivity and specificity of clinical criteria ≥2 points were 0.74 and 0.54, respectively. Supplemental Table S2 shows patient characteristics according to the criteria developed.

### 4.3. Criteria Validation

In MDV data, 90,870 patients were admitted to the ICU and discharged from hospital between 1 April 2014 and 31 October 2020. After exclusion, 15,302 eligible patients were included in the external validation cohort, and 4492 had PICS (BI < 70 or in-hospital death) (Figure 1). Regarding patient characteristics, patients in the MDV data were more likely to have a less severe condition and to be postoperative patients than those in the data of the Hitachi General Hospital Emergency and Critical Care Center (Supplemental Table S3).

**Figure 4:**
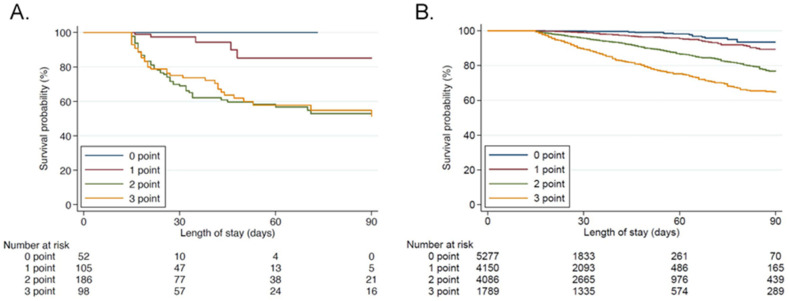
Kaplan Meier curve with each criterion point. The significance of differences is evaluated with the Log-rank test. (**A**): derivation cohort, (**B**): validation cohort.

In the external validation cohort, the AUROC of clinical criteria was 0.71 (95%CI 0.70–0.71), which was similar to the derivation cohort. The sensitivity and specificity of clinical criteria ≥2 points were 0.71 and 0.68, respectively (Table 2). Patient characteristics across the clinical criteria developed in the validation cohort are shown in Table 3. In all thresholds, higher severity, the more frequent use of mechanical ventilation and blood purification, higher mortality, longer ICU and hospital stays, and lower BI at discharge were observed in the PICS group with significance. These results were similar to those in the derivation cohort. Survival curves for patients with each point for the criteria are depicted by the Kaplan Meier method both in the derivation and validation cohorts in Figure 4. Survival time was significantly lower with criterion point increases with the Log-rank test (*p* < 0.001) in both groups.

### 4.4. Sensitivity Analysis

In the sensitivity analysis, we selected the optimal cut-off values for biomarkers using the six best machine-learning models only. Among these models, the XGBoost model had the highest performance for predicting PICS (Supplemental Table S4). Based on these models, similar to the primary analysis, a CRP of 2.0 mg/dL, albumin of 3.0 g/dL, and a lymphocyte count of 800/μL were the optimal cut-off values for PICS.

Regarding the use of the outcome BI < 60 and in-hospital death as the definition of PICS, optimal cut-off values for biomarkers were a CRP of 2.0 mg/dL, albumin of 3.0 g/dL, and a lymphocyte count of 800/μL, which were similar to those using the outcome BI < 70 and in-hospital death (Supplemental Figure S2). The AUROC of the criteria was 0.71 (95%CI 0.71–0.72), with sensitivity of 0.64 and specificity of 0.71 for clinical criteria ≥2 points in the external validation cohort (Supplemental Table S5).

Similarly, when using the outcome of BI < 80 and in-hospital death as the definition of PICS, the optimal cut-off values for biomarkers were CRP of 2.0 mg/dL, albumin of 3.0 g/dL, and a lymphocyte count of 800/μL, which were equivalent to those using the outcome of BI < 70 and in-hospital death (Supplemental Figure S3). The AUROC of the criteria was 0.70 (95%CI 0.69–0.71), with a sensitivity of 0.72 and a specificity of 0.68 for clinical criteria ≥2 points in the external validation cohort (Supplemental Table S6).

## 5. Discussion

We developed and validated clinical criteria for PICS based on the optimal cut-off values for CRP, albumin, and lymphocyte count using machine-learning approaches. The best cut-off values for the clinical criteria for PICS were CRP of 2.0 mg/dL, albumin of 3.0 g/dL, and a lymphocyte count of 800/μL. Using the criteria developed, the discrimination ability of the optimal cut-off values showed an AUROC of 0.71 in the validation cohort. Similar results were obtained using other definitions of BI < 60 or <80.

To the best of our knowledge, this is the first study to identify appropriate cut-off values for CRP, albumin, and lymphocyte count for PICS clinical criteria [7,10]. The strengths and novelty of the present study were the use of multiple models, the integration of the results of three biomarkers, and the provision of a clinical basis for their cut-off values in PICS criteria. Based on the present results, we propose the following PICS clinical criteria: hospital stay >14 days from ICU admission and CRP > 2.0 mg/dL, albumin >3.0 g/dL, and a total lymphocyte count <800/μL on day 14 of admission. Since these biomarkers are generally evaluated in clinical practice, these criteria may be easily implemented in clinical settings to identify patients with PICS. The criteria developed will identify patients with PICS in whom long-term mortality will be poor and activity of daily living will be declined.

Furthermore, the establishment of PICS clinical criteria will contribute to further improvements in clinical practice and future studies on PICS. Unfortunately, there is currently no established treatment to prevent or treat PICS [10,21]. However, excessive inflammation, malnutrition, and anemia are regarded as intervenient factors for the development of PICS, which may be treated using anti-inflammatory agents, nutrition therapy, exercise, or erythropoietin, thereby preventing PICS [22]. MDSCs or other biomarkers for immunosuppression of PICS could not be evaluated easily, therefore, we could not identify the PICS outcome and conduct clinical studies of PICS. By using the PICS clinical criteria developed in the present study, not only risk factors but also a number of interventions can be evaluated with the PICS outcome and discussed in clinical studies [23].

The appropriate cut-off values for albumin and lymphocyte count in the present study were similar to the proposed criteria with expert knowledge [7,10]. Previous studies showed that an albumin level lower than 3.0 to 3.5 g/dL was associated with increased mortality and the risk of infection in critically ill patients [24] as well as perioperative patients [25]. A lymphocyte count <700–800/μL was identified as a risk factor for mortality and infection under septic conditions [26,27]. Therefore, the use of albumin <3.0 g/dL and a lymphocyte count <800/μL as the criteria for PICS is clinically reasonable. The proposed cut-off value for CRP > 0.15 mg/dL was markedly lower than that in previous studies that predicted mortality and infectious complications [28,29]. CRP has been shown to converge at >3.0 mg/dL in patients with PICS [12]; therefore, the cut-off value for CRP > 2.0 mg/dL appears to be optimal for the PICS clinical criteria.

The condition of PICS is complicated, as described in the concept article [7,10]. If the main contributing factor to the development of PICS involves immunosuppression by the expansion of MDSCs, they need to be evaluated with clinical criteria in future studies; however, it is important to note that difficulties are associated with assessing MDSCs in clinical practice.

The present study had some limitations. BI is widely used as a surrogate outcome for PICS, but may not accurately evaluate this condition [14,19]. BI is often affected by factors other than PICSs, particularly cerebrovascular events; the number of stroke patients was higher in the BI < 70 group in the present study. Furthermore, although we excluded patients from nursing homes or those with long-term care insurance or home health care, eligible patients may have included those with low BI before admission. Moreover, we included patients who were hospitalized for >14 days in this analysis. However, some PICS patients who had not achieved full disease recovery may have been included in the population discharged within 14 days of admission. We excluded patients with long-term care insurance or home health care to identify those with newly occurring ADL decline. In the health care system in Japan, hospital stays are prolonged to achieve full disease recovery in patients without long-term care insurance or home health care. Therefore, this population may be more appropriate for the present study. However, since albumin on admission was lower in the ADL decline group, there may have been a risk of sarcopenia or malnutrition at baseline. The majority of patients were discharged from ICU in the present study. However, patients are not often treated in ICU for a full 14 days and treatments are continued after ICU discharge in the health care system in Japan [30]. Another limitation is that we exhaustively searched for optimal cut-off values using machine-learning approaches. The current methods may have been too explorative. However, the methodology of selecting cut-off values was non- arbitrary with the machine-learning approaches and matched the clinical interpretation. In addition, we did not evaluate other biomarkers for catabolism proposed in the literature, such as weight loss, body mass index, creatinine height index, pre-albumin, or retinol-binding protein.

## 6. Conclusions

We propose PICS clinical criteria with CRP < 2.0 mg/dL, albumin < 3.0 g/dL, and a lymphocyte count < 800/μL as the optimal cut-off values. The criteria developed will identify patients with PICS who have poor long-term mortality and activity in their daily lives.

## Figures and Tables

**Table 1:** Patient characteristics in the derivation cohort according to the PICS status.

	Overall	Non-PICS (Barthel Index ≥ 70 at Discharge)	PICS (Barthel Index < 70 or In-Hospital Death)	
Variables	*n* = 441	*n* = 150	*n* = 291	*p* Value
Age	71.1 ± 15.5	64.4 ± 16.6	74.6 ± 13.7	<0.001
Age ≥ 75, n (%)	235 (53.4)	52 (34.7)	183 (63.1)	<0.001
Male, n (%)	282 (64.0)	103 (68.7)	179 (61.5)	0.14
SOFA score on admission	6 (3, 9)	5 (2, 8)	6 (4, 9)	0.008
APACHEII score on admission	17 (12, 22)	14 (11, 21)	18 (12, 23)	0.002
Mechanical ventilation, n (%)	206 (46.7)	57 (38.0)	149 (51.2)	0.008
Duration (days)	9 (4, 18)	6 (3, 10.5)	10 (5, 25.5)	<0.001
Blood purification, n (%)	98 (22.2)	19 (12.7)	79 (27.2)	<0.001
Duration (days)	5 (2, 15)	3 (2, 8)	5 (3, 15)	0.31
Extracorporeal membrane oxygenation	10 (2.3)	5 (3.3)	5 (1.7)	0.29
Duration (days)	4.5 (2.8, 15)	4 (1.5, 5.5)	14 (3.5, 18.5)	0.17
Basic diseases on admission				
Sepsis n (%)	151 (34.2)	45 (30.0)	106 (36.5)	0.18
Cardiac failure, n (%)	67 (15.2)	26 (17.3)	41 (14.1)	0.37
Renal failure, n (%)	73 (16.6)	20 (13.3)	53 (18.2)	0.19
Respiratory failure, n (%)	58 (13.2)	16 (10.7)	42 (14.4)	0.26
Stroke, n (%)	42 (9.5)	5 (3.3)	37 (12.7)	0.0015
Endocrine and metabolic disorder, n (%)	69 (15.6)	25 (16.7)	44 (15.1)	0.67
Trauma, n (%)	67 (15.2)	25 (16.7)	42 (14.4)	0.54
Post-scheduled operation, n (%)	24 (5.4)	8 (5.3)	16 (5.5)	0.94
Mortality, n (%)	100 (22.7)	0 (0)	100 (34.4)	<0.001
Day on which patients died, days	21 (17, 33.8)		21 (17, 33.8)	
Length of ICU stay, days	9 (5, 14)	8 (5, 11)	10 (6, 16)	0.001
Length of hospital stay, days	26 (18, 45.5)	26 (17, 43.5)	26 (18, 47)	0.97
Barthel index at hospital discharge	55 (10, 100)	100 (90, 100)	15 (0, 40)	<0.001
Laboratory findings on day 1				
CRP (mg/dL)	2.9 (0.4, 12)	2.0 (0.2, 16.6)	3.7 (0.6, 10.5)	0.73
Albumin (g/dL)	3.2 ± 0.8	3.3 ± 0.9	3.1 ± 0.8	0.015
Lymphocytes (/μL)	1071 (574, 1852)	1092 (614, 2102)	1056 (544, 1764)	0.23
Laboratory findings on day 14				
CRP (mg/dL)	3.3 (1.1, 7.3)	1.8 (0.6, 5.7)	4.0 (1.5, 7.8)	<0.001
Albumin (g/dL)	2.5 ± 0.6	2.7 ± 0.6	2.4 ± 0.5	<0.001
Lymphocytes (/μL)	1080 (729, 1441)	1180 (908, 1547)	1008 (660, 1330)	<0.001

Abbreviations: PICS, persistent inflammation, immunosuppression, and catabolism syndrome; SOFA, sequential organ failure assessment; APACHE II, acute physiology, chronic health evaluation; ICU, intensive care unit; CRP, C-reactive protein.

**Table 2:** Predictive ability of criteria developed for PICS in derivation and validation cohorts.

	Derivation Cohort			Validation Cohort		
	AUROC	Sensitivity	Specificity	AUROC	Sensitivity	Specificity
Discrimination ability	0.67	-	-	0.71	-	-
Sum of points in criteria						
1	-	0.94	0.23	-	0.85	0.43
2	-	0.74	0.54	-	0.62	0.71
3	-	0.27	0.88	-	0.24	0.93

One point is given when any of the following items are positive: C-reactive protein > 2.0 mg/dL, albumin < 3.0 g/dL, or a lymphocyte count < 800/μL. Abbreviations: PICS, persistent inflammation, immunosuppression, and catabolism syndrome; AUROC, area under the receiver operating characteristic.

**Table 3:** Patient characteristics according to PICS criteria developed in the validation cohort.

	Sum of Points in PICS Criteria (One Point Is Given When Any of the Following Items Are Positive: CRP > 2.0 mg/dL, Albumin < 3.0 g/dL, or a Lymphocyte Count < 800/μL)								
	0	≥1		<2	≥2		<3	3	
Variables	*n* = 5277	*n* = 10,025	*p* Value	*n* = 9427	*n* = 5875	*p* Value	*n* = 13,513	*n* = 1789	*p* Value
Age	68.0 (13.4)	72.0 (12.4)	<0.001	69.3 (13.2)	72.8 (12.1)	<0.001	70.2 (12.9)	74.0 (11.7)	<0.001
Age ≥ 75, n (%)	1803 (34.2)	4817 (48.0)	<0.001	3659 (38.8)	2961 (50.4)	<0.001	5645 (41.8)	975 (54.5)	<0.001
Male, n (%)	3235 (61.3)	6416 (64.0)	0.001	5796 (61.5)	3855 (65.6)	<0.001	8511 (63.0)	1140 (63.7)	0.54
SOFA on admission	3 (1–4)	4 (1–6)	<0.001	3 (1–5)	4 (2–7)	<0.001	3 (1–5)	5 (2–7)	<0.001
Mechanical ventilation, n (%)	1086 (20.6)	4169 (41.6)	<0.001	2414 (25.6)	2841 (48.4)	<0.001	4248 (31.4)	1007 (56.3)	<0.001
Duration (days)	1 (1–4)	4 (1–15)	<0.001	2 (1–5)	7 (2–21)	<0.001	3 (1–8)	12 (3–28)	<0.001
Blood purification, n (%)	154 (2.9)	1470 (14.7)	<0.001	498 (5.3)	1126 (19.2)	<0.001	1138 (8.4)	486 (27.2)	<0.001
Duration (days)	7 (3–19)	9 (4–22)	0.012	7 (3–22)	10 (4–21)	0.007	8 (4–21)	11 (5–21)	0.003
Extracorporeal membrane oxygenation	9 (0.2)	127 (1.3)	<0.001	25 (0.3)	111 (1.9)	<0.001	89 (0.7)	47 (2.6)	<0.001
Duration (days)	1 (1–1)	1 (1–4)	0.090	1 (1–1)	1 (1–4)	0.047	1 (1–2)	1 (1–7)	0.003
Basic diseases on admission									
Sepsis n (%)	221 (4.2)	1312 (13.1)	<0.001	585 (6.2)	948 (16.1)	<0.001	1206 (8.9)	327 (18.3)	<0.001
Cardiac failure, n (%)	1476 (28.0)	2082 (20.8)	<0.001	2445 (25.9)	1113 (18.9)	<0.001	3257 (24.1)	301 (16.8)	<0.001
Renal failure, n (%)	31 (0.6)	281 (2.8)	<0.001	125 (1.3)	187 (3.2)	<0.001	240 (1.8)	72 (4.0)	<0.001
Respiratory failure, n (%)	138 (2.6)	800 (8.0)	<0.001	364 (3.9)	574 (9.8)	<0.001	727 (5.4)	211 (11.8)	<0.001
Stroke, n (%)	392 (7.4)	488 (4.9)	<0.001	607 (6.4)	273 (4.6)	<0.001	806 (6.0)	74 (4.1)	0.002
Endocrine and metabolic disorder, n (%)	70 (1.3)	137 (1.4)	0.84	142 (1.5)	65 (1.1)	0.037	189 (1.4)	18 (1.0)	0.18
Trauma, n (%)	256 (4.9)	540 (5.4)	0.16	446 (4.7)	350 (6.0)	<0.001	696 (5.2)	100 (5.6)	0.43
Post-scheduled operation, n (%)	2871 (54.4)	4078 (40.7)	<0.001	4867 (51.6)	2082 (35.4)	<0.001	6369 (47.1)	580 (32.4)	<0.001
Mortality, n (%)	27 (0.5)	1034 (10.3)	<0.001	143 (1.5)	918 (15.6)	<0.001	603 (4.5)	458 (25.6)	<0.001
Day on which patients died, days	5 (1–13)	13 (5–14)	0.006	9 (3–14)	13 (5–14)	0.009	11 (4–14)	14 (5–14)	0.027
Length of ICU stay, days	2 (1–4)	3 (1–8)	<0.001	2 (1–4)	4 (2–12)	<0.001	2 (1–5)	6 (2–14)	<0.001
Length of hospital stay, days	25 (19–34)	34 (24–53)	<0.001	26 (20–37)	39 (26–61)	<0.001	29 (21–43)	43 (29–68)	<0.001
Barthel index at hospital discharge	100 (100–100)	100 (55–100)	<0.001	100 (95–100)	100 (30–100)	<0.001	100 (85–100)	85 (15–100)	<0.001
Laboratory findings on day 1									
CRP (mg/dL)	3.9 (1.6–7.1)	6.4 (3.3–11.0)	<0.001	4.7 (2.1–8.0)	7.0 (3.6–12.8)	<0.001	5.2 (2.5–8.9)	7.8 (4.0–14.6)	<0.001
Albumin (g/dL)	3.1 (0.4)	2.8 (0.6)	<0.001	3.0 (0.5)	2.7 (0.6)	<0.001	2.9 (0.5)	2.6 (0.6)	<0.001
Lymphocytes (/μL)	1069 (772–1429)	801 (536–1162)	<0.001	993 (700–1356)	736 (488–1082)	<0.001	945 (648–1307)	583 (383–833)	<0.001
Laboratory findings on day 14									
CRP (mg/dL)	0.5 (0.2–1.0)	3.5 (1.7–7.1)	<0.001	0.8 (0.3–1.7)	5.4 (3.1–9.5)	<0.001	1.4 (0.5–3.7)	7.1 (4.3–12.3)	<0.001
Albumin (g/dL)	3.5 (0.3)	2.7 (0.5)	<0.001	3.3 (0.5)	2.4 (0.5)	<0.001	3.0 (0.6)	2.2 (0.4)	<0.001
Lymphocytes (/μL)	1490 (1190–1884)	1043 (739–1418)	<0.001	1385 (1088–1785)	856 (624–1225)	<0.001	1287 (994–1682)	578 (429–700)	<0.001

Abbreviations: CRP, C-reactive protein; SOFA, sequential organ failure assessment; ICU, intensive care unit.

## Data Availability

The datasets analyzed during the present study are not publicly available due to contracts with Medical Data Vision claims.

## Supplementary Materials

The following supporting information can be downloaded at: https://www.mdpi.com/article/10.3390/jcm11195790/s1, Figure S1: Changes in AUROC for each combination of the order of biomarkers in machine-learning models; Figure S2: Scatter plots of cut-off values for biomarkers and corresponding AUROCs in 36 models using the outcome of Barthel index < 60; Figure S3: Scatter plots of cut-off values for biomarkers and corresponding AUROCs in 36 models using the outcome of Barthel index < 80; Table S1: Variables for missing estimations using XGBoost; Table S2: Patient characteristics according to criteria developed in the derivation cohort; Table S3: Patient characteristics in the validation cohort; Table S4: The best model for predicting persistent inflammation, immunosuppression, and catabolism syndrome (Barthel index < 70 or in-hospital death) in each machine-learning model using C-reactive protein, albumin, and lymphocyte count; Table S5: Predictive ability of criteria developed for PICS (Barthel index < 60 or in-hospital death) in derivation and validation cohorts; Table S6: Predictive ability of criteria developed for PICS (Barthel index < 80 or in-hospital death) in derivation and validation cohorts.
