# Supplementary material for: Clinical Criteria for Persistent Inflammation, Immunosuppression, and Catabolism Syndrome: An Exploratory Analysis of Optimal Cut-Off Values for Biomarkers
Source: J Clin Med. 2022 Sep 29;11(19):5790. doi: 10.3390/jcm11195790 (PMC9571101; doi:10.3390/jcm11195790)
Supplement: Supplementary file 1 [file jcm-11-05790-s001.zip › Supplemental Table S1.pdf]

Supplemental Table S1. Variables for missing estimations using XGBoost.

	**Variables**
Complete blood count	White blood cells, hemoglobin, hematocrit, basophils, eosinophils, segmented cells
Coagulation markers	Platelets, D-dimer, FDP, the prothrombin ratio
Biochemistry	Lactate, total protein, C-reactive protein, albumin, lymphocytes

Abbreviations: FDP, fibrin／fibrinogen degradation products
