# Supplementary material for: Clinical Criteria for Persistent Inflammation, Immunosuppression, and Catabolism Syndrome: An Exploratory Analysis of Optimal Cut-Off Values for Biomarkers
Source: J Clin Med. 2022 Sep 29;11(19):5790. doi: 10.3390/jcm11195790 (PMC9571101; doi:10.3390/jcm11195790)
Supplement: Supplementary file 1 [file jcm-11-05790-s001.zip › Supplemental Table S2.pdf]

Supplemental Table S2. Patient characteristics according to criteria developed in the derivation cohort

	Sum of points in developed criteria (one point is given when any of the following items are positive: CRP >2.0 mg/dl, albumin <3.0 g/dl, or a lymphocyte count <800/mm^3)								
	**0**	**≥1**		**<2**	**≥2**		**<3**	**3**	
**Variables**	**n=52**	**n=389**	**P value**	**n=157**	**n=284**	**P value**	**n=343**	**n=98**	**P value**
**Age**	65.6±17.7	71.9±15.1	0.0057	68.6±17.9	72.6±13.9	0.010	70.6±16.4	73.2±16.4	0.14
**Age ≥75, n (%)**	21 (40.4)	214 (55.2)	0.045	75 (48.1)	160 (56.3)	0.097	179 (52.3)	56 (57.1)	0.40
**Male, n (%)**	21 (40.4)	138 (34.5)	0.49	61 (38.9)	98 (34.5)	0.36	126 (36.7)	33 (33.7)	0.58
**SOFA**	4.5 (2, 7)	6 (3, 9)	0.0019	4 (2, 7.8)	7 (4, 10)	<0.0001	5 (3, 8.75)	8 (4, 11)	<0.0001
**APACHEII**	12.5 (10, 19)	17 (12, 23)	0.0005	15 (11, 20)	17.5 (13, 23)	0.0015	16 (11, 21)	19 (14, 26)	0.0021
**Mechanical ventilation, n (%)**	17 (32.7)	189 (48.6)	0.029	47 (29.9)	159 (56.0)	<0.0001	136 (49.7)	70 (71.4)	<0.0001
**Duration (days)**	5 (3, 6)	9 (5, 20)	0.0020	6 (4, 11)	10 (5, 23)	0.029	8.5 (5, 22)	9 (4, 16)	0.60
**Blood purification, n (%)**	3 (5.8)	95 (24.4)	0.0006	13 (8.3)	85 (29.9)	<0.0001	61 (17.8)	37 (37.8)	<0.0001
**Duration (days)**	3 (3, 3)	5 (2, 15)	0.58	3 (2, 11)	5 (2.5, 15)	0.28	4 (2, 13)	7 (3, 17)	0.17
**Extracorporeal membrane oxygenation**	0 (0)	10 (2.6)	0.11	2 (1.3)	8 (2.8)	0.28	8 (2.3)	2 (2.0)	0.86
**Duration (days)**	0 (0)	4.5 (2.8, 15)	1	3 (2, 4)	5.5 (3.3, 17)	0.29	5.5 (4, 17)	2 (1, 3)	0.089
**Basic diseases on admission**									
**Sepsis n (%)**	6 (11.5)	145 (37.3)	<0.0001	45 (28.7)	106 (37.3)	0.065	113 (32.9)	38 (38.8)	0.29
**Cardiac failure, n (%)**	10 (19.2)	57 (14.7)	0.40	24 (15.3)	43 (15.1)	0.97	52 (15.1)	15 (15.3)	0.97
**Renal failure, n (%)**	5 (9.6)	68 (17.5)	0.13	22 (14.1)	51 (18.0)	0.28	50 (14.6)	23 (23.5)	0.043
**Respiratory failure, n (%)**	6 (11.5)	52 (13.4)	0.71	20 (12.7)	38 (13.4)	0.85	44 (12.8)	14 (14.3)	0.71
**Stroke, n (%)**	2 (3.9)	40 (10.3)	0.10	14 (8.9)	28 (9.9)	0.75	32 (9.3)	10 (10.2)	0.80
**Endocrine and metabolic disorder, n (%)**	9 (17.3)	60 (15.4)	0.73	21 (13.4)	48 (16.9)	0.32	50 (14.6)	19 (19.4)	0.26
**Trauma, n (%)**	14 (26.9)	53 (13.6)	0.019	28 (17.8)	39 (13.7)	0.25	50 (14.6)	17 (17.4)	0.51
**Post-scheduled operation, n (%)**	0 (0)	24 (6.2)	0.013	4 (2.6)	20 (7.0)	0.035	16 (4.7)	8 (8.2)	0.20
**Mortality, n (%)**	0 (0)	100 (25.7)	<0.0001	5 (3.2)	95 (33.5)	<0.0001	63 (18.4)	37 (37.8)	<0.0001
**Day on which patients died, days**		21 (17, 33.8)		35 (18.5, 47)	21 (17, 32)	0.26	21 (17, 32)	20 (16.5, 42.5)	0.70
**Length of ICU stay, days**	6 (4, 9)	9 (5.5, 15)	0.0060	6 (4.3, 10)	10 (6, 15.8)	<0.0001	8 (5, 13)	13 (8, 17)	<0.0001
**Length of hospital stay, days**	20 (16, 28)	27 (18, 48)	0.0021	23 (18, 35)	27.5 (18, 53)	0.037	25 (17, 39)	36 (20, 60.0)	0.0003

**Barthel index at hospital discharge**	100 (50, 100)	10 (50, 95)	<0.0001	75 (20, 100)	40 (5, 90)	<0.0001*	65 (15, 100)	20 (0, 82.5)	0.0018
**Laboratory findings on day 1**									
**CRP (mg/dl)**	0.7 (0.1, 1.6)	4.9 (0.4, 14.1)	<0.0001	1.6 (0.4, 7.9)	5.0 (0.3, 14.4)	0.014	2.3 (0.3, 10.7)	5.9 (0.4, 17.0)	0.056
**Albumin (g/dl)**	3.8±0.5	3.1±0.8	<0.0001	3.4±0.8	3.0±0.8	<0.0001	3.2±0.8	2.9±0.8	0.0029
**Lymphocytes (/μl)**	1268 (776, 2125)	1056 (555, 1798)	0.045	1002 (723, 2110)	1003 (514, 1773)	0.013	1110 (663, 2006)	765 (372, 1484)	0.0004
**Laboratory findings on day 14**									
**CRP (mg/dl)**	0.5 (0.2, 0.9)	4.0 (1.6, 7.7)	<0.0001	0.7 (0.4, 1.4)	6.3 (3.3, 9.1)	<0.0001	2.2 (0.7, 6.3)	7.4 (3.9, 11.4)	<0.0001
**Albumin (g/dl)**	3.4±0.3	2.4±0.5	<0.0001	2.9±0.49	2.2±0.44	<0.0001	2.6±5.4	2.1±0.4	<0.0001
**Lymphocytes (/μl)**	1494 (1144, 1828)	1008 (687, 1337)	<0.0001	1342 (1093, 1732)	869 (584, 1220)	<0.0001	1215 (965, 1554)	538 (374, 670)	<0.0001

Abbreviations: CRP, C-reactive protein; SOFA, sequential organ failure assessment; APACHE II, acute physiology, chronic health evaluation; ICU, intensive care unit
