# Supplementary material for: Clinical Criteria for Persistent Inflammation, Immunosuppression, and Catabolism Syndrome: An Exploratory Analysis of Optimal Cut-Off Values for Biomarkers
Source: J Clin Med. 2022 Sep 29;11(19):5790. doi: 10.3390/jcm11195790 (PMC9571101; doi:10.3390/jcm11195790)
Supplement: Supplementary file 1 [file jcm-11-05790-s001.zip › Supplemental Table S3.pdf]

Supplemental Table S3. Patient characteristics in the validation cohort

Variables	Overall n=15,302	Barthel index ≥70 at discharge n=10,810	Barthel index <70 or in-hospital death n=4,492	P value
**Age**	70.6 (12.9)	68.4 (12.4)	75.9 (12.4)	<0.001
**Age ≥75, n (%)**	6,620 (43.3)	3,793 (35.1)	2,827 (62.9)	<0.001
**Male, n (%)**	9,651 (63.1)	7,053 (65.2)	2,598 (57.8)	<0.001
**SOFA score on admission**	3 (1-5)	3 (1-5)	5 (3-7)	<0.001
**Mechanical ventilation, n (%)**	5,255 (34.3)	3,078 (28.5)	2,177 (48.5)	<0.001
**Duration (days)**	3 (1-12)	2 (1-5)	11 (3-28)	<0.001
**Blood purification, n (%)**	1,624 (10.6)	707 (6.5)	917 (20.4)	<0.001
**Duration (days)**	9 (4-21)	7 (3-20)	11 (5-22)	<0.001
**Extracorporeal membrane oxygenation**	136 (0.9)	57 (0.5)	79 (1.8)	<0.001
**Duration (days)**	1 (1-3)	1 (1-2)	1 (1-4)	0.15
**Basic diseases on admission**				
**Sepsis n (%)**	1,533 (10.0)	785 (7.3)	748 (16.7)	<0.001
**Cardiac failure, n (%)**	3,558 (23.3)	2,718 (25.1)	840 (18.7)	<0.001
**Renal failure, n (%)**	312 (2.0)	134 (1.2)	178 (4.0)	<0.001
**Respiratory failure, n (%)**	938 (6.1)	372 (3.4)	566 (12.6)	<0.001
**Stroke, n (%)**	880 (5.8)	239 (2.2)	641 (14.3)	<0.001
**Endocrine and metabolic disorder, n (%)**	207 (1.4)	117 (1.1)	90 (2.0)	<0.001
**Trauma, n (%)**	796 (5.2)	330 (3.1)	466 (10.4)	<0.001
**Post-scheduled operation, n (%)**	6,949 (45.4)	6,330 (58.6)	619 (13.8)	<0.001
**Mortality, n (%)**	1,061 (6.9)	0 (0.0)	1,061 (23.6)	<0.001
**Day on which patients died, days**	13 (4-14)		13 (4-14)	
**Length of ICU stay, days**	3 (1-6)	2 (1-4)	5 (2-13)	<0.001
**Length of hospital stay, days**	30 (22-46)	28 (21-40)	38 (25-62)	<0.001
**Barthel index at hospital discharge**	100 (75-100)	100 (100-100)	20 (0-50)	<0.001
**Laboratory findings on day 1**				
**CRP (mg/dl)**	5.4 (2.6-9.3)	5.2 (2.6-8.5)	6.6 (2.8-13.7)	<0.001
**Albumin (g/dl)**	2.9 (0.6)	3.0 (0.5)	2.7 (0.6)	<0.001
**Lymphocytes (/µl)**	898 (604-1264)	943 (647-1305)	769 (506-1140)	<0.001
**Laboratory findings on day 14**				
**CRP (mg/dl)**	1.8 (0.6-4.9)	1.4 (0.5-3.8)	3.2 (1.0-7.6)	<0.001

**Albumin (g/dl)**	2.9 (0.6)	3.1 (0.5)	2.5 (0.6)	<0.001
**Lymphocytes (/µl)**	1206 (870-1617)	1282 (960-1691)	1007 (684-1400)	<0.001

Abbreviations: SOFA, sequential organ failure assessment; ICU, intensive care unit; CRP, C-reactive protein
