# Supplementary material for: Clinical Criteria for Persistent Inflammation, Immunosuppression, and Catabolism Syndrome: An Exploratory Analysis of Optimal Cut-Off Values for Biomarkers
Source: J Clin Med. 2022 Sep 29;11(19):5790. doi: 10.3390/jcm11195790 (PMC9571101; doi:10.3390/jcm11195790)
Supplement: Supplementary file 1 [file jcm-11-05790-s001.zip › Supplemental Table S4.pdf]

Supplemental Table S4. The best model for predicting persistent inflammation, immunosuppression, and catabolism syndrome (Barthel index <70 or in-hospital death) in each machine-learning model using C-reactive protein, albumin, and the lymphocyte count

Model	Area under the receiver operating characteristic	C-reactive protein, mg/dl	Albumin, g/dl	Lymphocyte count, /mm^3
XGBoost	0.657	2.0	3.1	880
Logistic regression	0.641	1.9	3.0	600
Linear regression	0.647	2.0	3.0	740
Gaussian naïve Bayes	0.647	2.1	2.9	600
Ridge regression	0.647	2.1	3.0	740
Random forest	0.616	2.0	3.6	820

Persistent inflammation, immunosuppression, and catabolism syndrome was defined as Barthel index <70 at discharge.
