# Supplementary material for: Clinical Criteria for Persistent Inflammation, Immunosuppression, and Catabolism Syndrome: An Exploratory Analysis of Optimal Cut-Off Values for Biomarkers
Source: J Clin Med. 2022 Sep 29;11(19):5790. doi: 10.3390/jcm11195790 (PMC9571101; doi:10.3390/jcm11195790)
Supplement: Supplementary file 1 [file jcm-11-05790-s001.zip › Supplemental Table S5.pdf]

Supplemental Table S5. Predictive ability of criteria developed for PICS (Barthel index <60 or in-hospital death) in derivation and validation cohorts

	Derivation cohort			Validation cohort		
	AUROC	Sensitivity	Specificity	AUROC	Sensitivity	Specificity
Discrimination ability	0.67	-	-	0.71	-	-
Sum of points in criteria						
1	-	0.94	0.21	-	0.86	0.42
2	-	0.75	0.53	-	0.64	0.71
3	-	0.29	0.88	-	0.25	0.93

One point is given when any of the following items are positive: CRP >2.0 mg/dl, albumin <3.0 g/dl, or a lymphocyte count <800/mm^3

Abbreviations: PICS, persistent inflammation, immunosuppression, and catabolism syndrome; AUROC, area under the receiver operating characteristic
