# Supplementary material for: Clinical Criteria for Persistent Inflammation, Immunosuppression, and Catabolism Syndrome: An Exploratory Analysis of Optimal Cut-Off Values for Biomarkers
Source: J Clin Med. 2022 Sep 29;11(19):5790. doi: 10.3390/jcm11195790 (PMC9571101; doi:10.3390/jcm11195790)
Supplement: Supplementary file 1 [file jcm-11-05790-s001.zip › Supplemental Table S6.pdf]

Supplemental Table S6. Predictive ability of criteria developed for PICS (Barthel index <80 or in-hospital death) in derivation and validation cohorts

	Derivation cohort			Validation cohort		
	AUROC	Sensitivity	Specificity	AUROC	Sensitivity	Specificity
Discrimination ability	0.67	-	-	0.70	-	-
Sum of points in criteria						
1	-	0.94	0.24	-	0.84	0.43
2	-	0.73	0.55	-	0.60	0.72
3	-	0.27	0.88	-	0.23	0.94

One point is given when any of the following items are positive: CRP >2.0 mg/dl, albumin <3.0 g/dl, or a lymphocyte count <800/mm^3

Abbreviations: PICS, persistent inflammation, immunosuppression, and catabolism syndrome; AUROC, area under the receiver operating characteristic
