# Supplementary material for: Clinical Criteria for Persistent Inflammation, Immunosuppression, and Catabolism Syndrome: An Exploratory Analysis of Optimal Cut-Off Values for Biomarkers
Source: J Clin Med. 2022 Sep 29;11(19):5790. doi: 10.3390/jcm11195790 (PMC9571101; doi:10.3390/jcm11195790)
Supplement: Supplementary file 1 [file jcm-11-05790-s001.zip › Supplementary Figure S2.pdf]

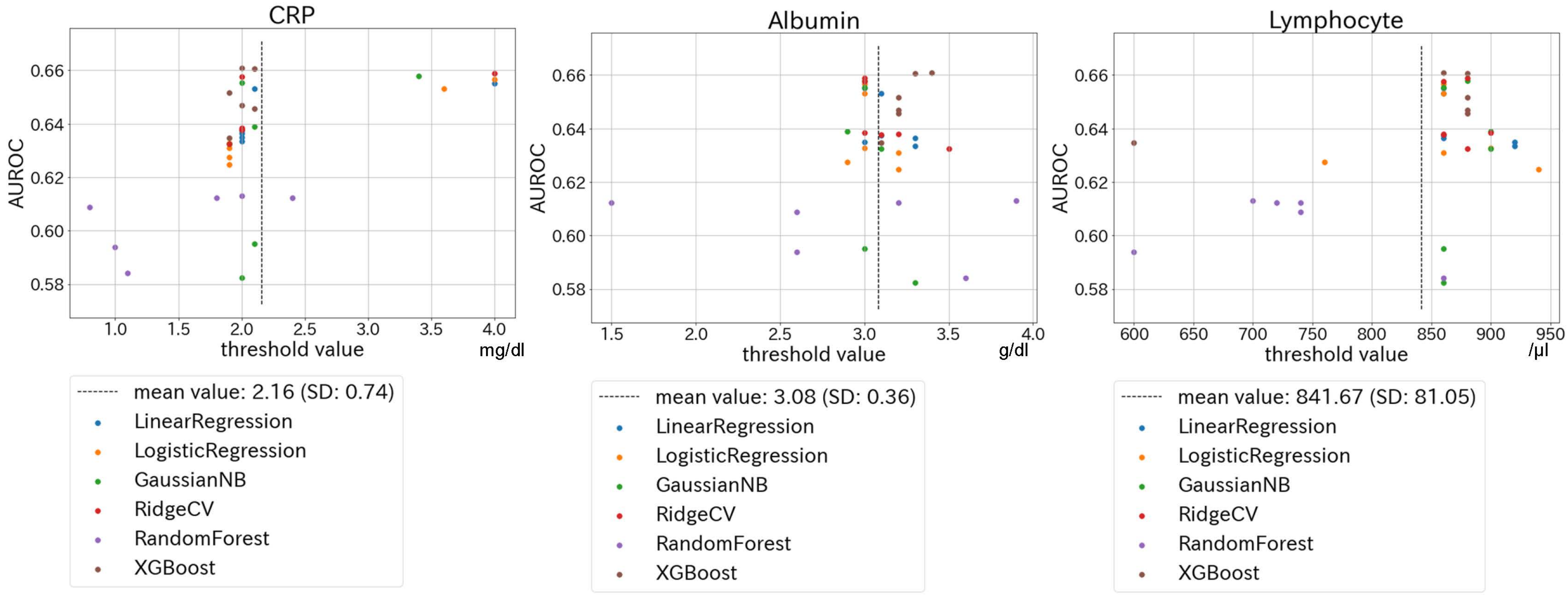

CRP
AUROC
threshold value
mg/dl
mean value: 2.16 (SD: 0.74)
LinearRegression
LogisticRegression
GaussianNB
RidgeCV
RandomForest
XGBoost
Albumin
AUROC
threshold value
g/dl
mean value: 3.08 (SD: 0.36)
LinearRegression
LogisticRegression
GaussianNB
RidgeCV
RandomForest
XGBoost
Lymphocyte
AUROC
threshold value
/μl
mean value: 841.67 (SD: 81.05)
LinearRegression
LogisticRegression
GaussianNB
RidgeCV
RandomForest
XGBoost
