# Supplementary figures and images for: Clinical Criteria for Persistent Inflammation, Immunosuppression, and Catabolism Syndrome: An Exploratory Analysis of Optimal Cut-Off Values for Biomarkers
Source: J Clin Med. 2022 Sep 29;11(19):5790. doi: 10.3390/jcm11195790 (PMC9571101; doi:10.3390/jcm11195790)

1. Logistic regression

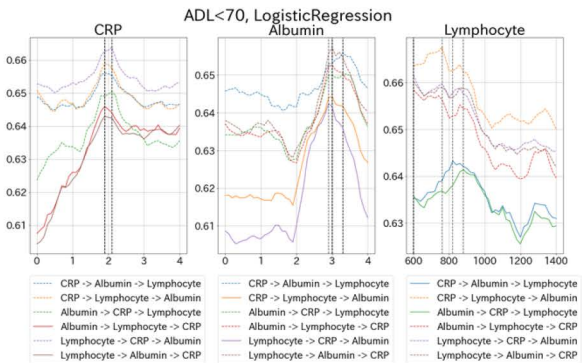

2. Linear regression

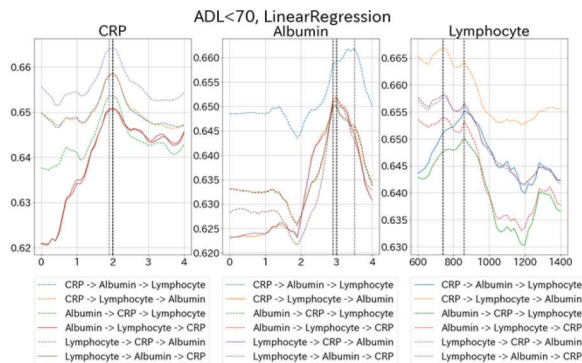

3. Gaussian naïve Bayes

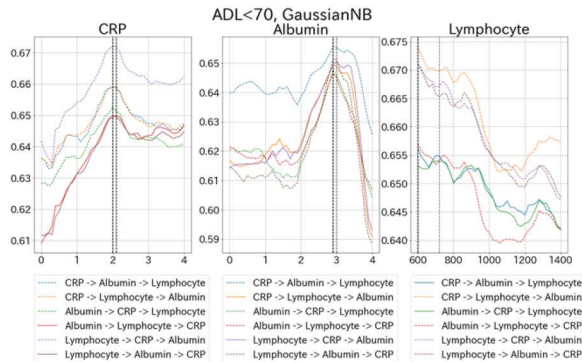

4. Ridge regression

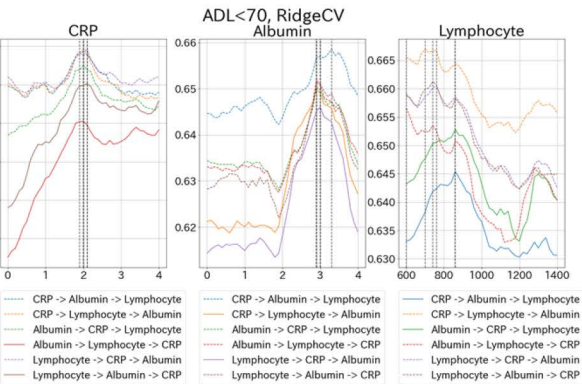

5. Random forest

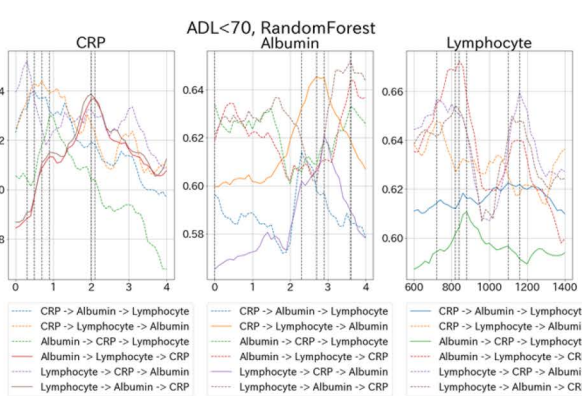

Supplement: Supplementary file 1 [file jcm-11-05790-s001.zip › Supplementary Figure S1.pdf]
