# Supplementary material for: Clinical Criteria for Persistent Inflammation, Immunosuppression, and Catabolism Syndrome: An Exploratory Analysis of Optimal Cut-Off Values for Biomarkers
Source: J Clin Med. 2022 Sep 29;11(19):5790. doi: 10.3390/jcm11195790 (PMC9571101; doi:10.3390/jcm11195790)
Supplement: Supplementary file 1 [file Supplementary Figure S3.pdf › Supplementary Figure S3.pdf]

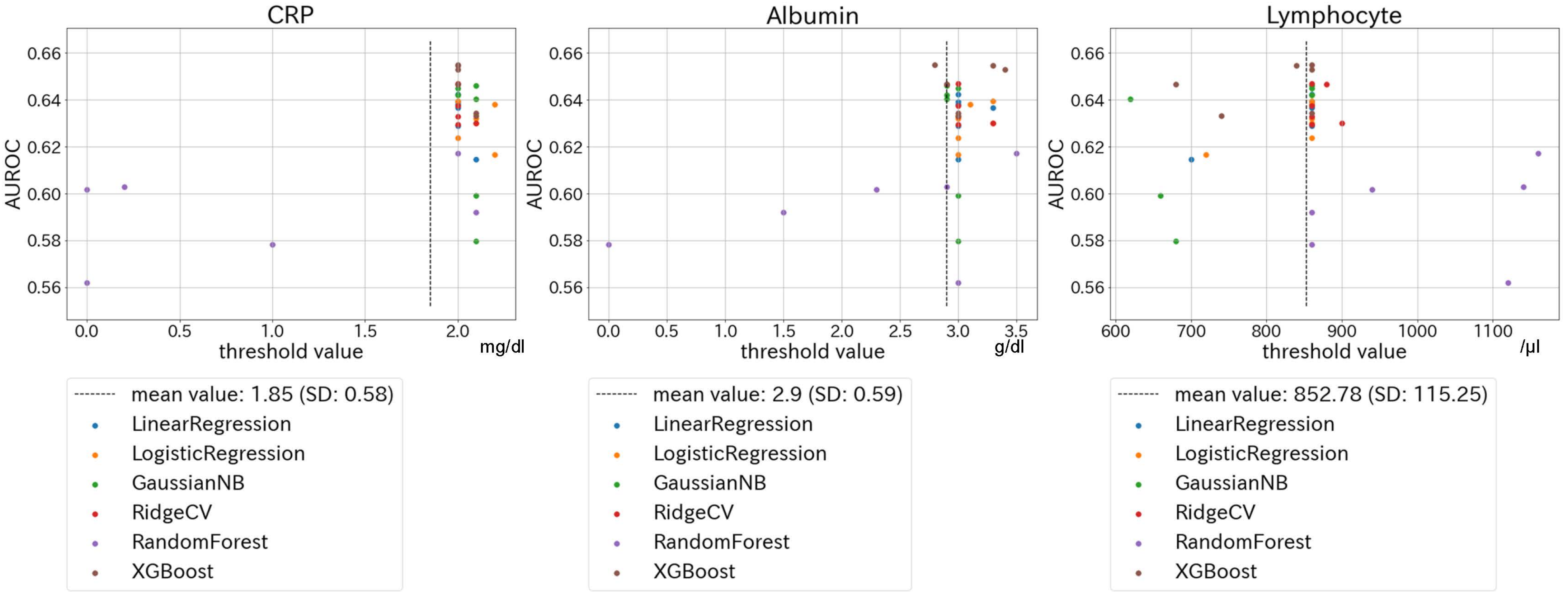
CRP
Albumin
Lymphocyte
AUROC
threshold value
mg/dl
g/dl
/μl
mean value: 1.85 (SD: 0.58)
mean value: 2.9 (SD: 0.59)
mean value: 852.78 (SD: 115.25)
LinearRegression
LogisticRegression
GaussianNB
RidgeCV
RandomForest
XGBoost
